# Kidney transplantation in systemic sclerosis: Advances in graft, disease, and patient outcome

**DOI:** 10.3389/fimmu.2022.878736

**Published:** 2022-07-26

**Authors:** Federica Maritati, Michele Provenzano, Sarah Lerario, Valeria Corradetti, Claudia Bini, Marco Busutti, Valeria Grandinetti, Vania Cuna, Gaetano La Manna, Giorgia Comai

**Affiliations:** Nephrology, Dialysis and Renal Transplant Unit, IRCCS—Azienda Ospedaliero-Universitaria di Bologna, Alma Mater Studiorum University of Bologna, Bologna, Italy

**Keywords:** systemic sclerosis, scleroderma renal crisis, ACE inhibitors, renal replacement therapy, kidney transplant

## Abstract

Systemic sclerosis (SSc) is an immune-mediated rheumatic disease characterized by vascular abnormalities, tissue fibrosis, and inflammation. Renal disease occurring in patients with SSc may have a variable clinicopathological picture. However, the most specific renal condition associated with this disease is the scleroderma renal crisis (SRC), characterized by acute onset of renal failure and severe hypertension. SRC develops in about 20% of cases of SSc, especially in those patients with diffuse cutaneous disease. The prognosis of this condition is often negative, with a rapid progression to end-stage renal disease (ESRD). The advent of the antihypertensive angiotensin-converting enzyme inhibitors in 1980 was associated with a significant improvement in patients’ survival and recovery of renal function. However, the prognosis of these patients can still be improved. The dialytic condition is associated with early death, and mortality is significantly higher than among patients undergoing renal replacement therapy (RRT) due to other conditions. Patients with SRC who show no signs of renal functional recovery despite timely blood pressure control are candidates for kidney transplantation (KT). In this review, we reported the most recent advances in KT in patients with ESRD due to SSc, with a particular overview of the risk of disease recurrence after transplantation and the evolution of other disease manifestations.

## Introduction

Systemic sclerosis (SSc) is a chronic condition characterized by vascular dysfunction, inflammation, excessive collagen accumulation, and progressive fibrosis of the skin and internal organs (1). It is also as scleroderma because of its clinical distinctive feature, namely, the thickening and hardening of the skin (2). SSc is a rare disease predominantly occurring in women with an annual incidence of 10–20 cases per million population and a prevalence of 30–300 per million population (3, 4). The pathogenesis involves a number of different mechanisms, including the contribution of genetic and environmental factors. For instance, the association between SSc and HLA DRB1 and DQB loci, PTPN22, NLRP1, STAT4, and IRF5 has been described (5). An emerging and interesting field is the study of epigenetics factors, like microRNA (miRNA). In scleroderma, they are involved in promoting microvascular changes (miRNA-22) and pro-fibrotic activation *via* toll-like receptor signaling (miRNA-21 and 155) (6). Among environmental factors, infectious agents seem to be able to promote the development of the disease, particularly viral agents such as Epstein–Barr virus, cytomegalovirus, human herpes virus 6, and parvovirus B19. These microorganisms may contribute to the onset of SSc by determining an increase in antibody and cell-mediated immune response and molecular mimicry. Different chemical agents have been proposed as potential stimulating factors of SSc, for example, organic solvents, pesticides, heavy metals, silicone breast implants, and silica (7).

Mediators of changes in a vascular tone such as endothelin-1, nitric oxide, and superoxide anions have been shown to trigger the development of SSc and also to anticipate other features of the disease (8, 9). The perpetual activation of endothelial cells amplifies the inflammatory stimuli with leukocyte migration out of the blood vessels (10). Moreover, it has been demonstrated that subpopulations of T lymphocytes (CD3 and CD4) have an increased ability to adhere to the endothelial cells (11). All these cell–cell and cell–matrix interactions lead, in turn, to the production of cytokines and growth factors, i.e., transforming growth factor-beta (TGF-β), interleukin (IL)-1, IL-4, IL-6, IL-8, IL-13, and CXCL4, which mediate the activation of fibroblasts and finally to the matrix production and deposition (12).

Almost all SSc patients have circulating autoantibodies against different antigens including topoisomerase I, centromere antigens, ribonucleic acid (RNA) polymerase III, PM-Scl, and fibrillin-1 (13). The mechanisms for the antibody synthesis in SSc are not well defined and are likely related to the generation of antigens from reactive oxygen species, which act as stimuli (14). In any case, the assessment of autoantibody levels may inform about the specific organ involvement in SSc. The anti-topoisomerase I antibodies correlate with diffuse cutaneous involvement and severe lung interstitial disease, whereas anti-centromere antibodies are highly associated with limited cutaneous involvement and with pulmonary arterial hypertension (15–17). Anti-RNA polymerase III antibodies are commonly detected in SSc patients with diffuse cutaneous involvement and have been shown to predict the development of scleroderma renal crisis (SRC) as well as have shown to be associated with malignancy and gastric antral vascular ectasias (18–20).

Apart from autoantibodies, a series of new and potential biomarkers have been explored. Among them, several cytokines (IL-6), chemokines (CXCL4–8–10), growth factors (VEGF and TGF-β), and other molecules (MMP7–9–12) can be treated and can reflect the fibrosis activity. More specific markers related to interstitial lung disease (ILD), for example, Krebs von den Lungen-6 (KL-6), which is increased in patients affected by ILD, and Surfactant Protein-A and D (SP-A, SP-D), which reflect the extent of damage to the capillary/alveolar barrier, are under study (21). Certainly, the potential biomarkers need to be validated and standardized in order to be used in clinical practice.

Clinical manifestations of SSc are generally severe and are associated with the degree of skin involvement (which affects almost all cases) and internal organ involvement. Skin involvement mainly affects hands, fingers, and faces in limited cutaneous systemic sclerosis; conversely, in diffuse cutaneous disease in addition to puffy hands, trunk and proximal arms and thighs are involved. The internal organ involvement can include digital vasculopathy, gastrointestinal, musculoskeletal, lung, and kidney manifestations, and in men, it can be associated with sexual dysfunction (3, 22, 23). With respect to cutaneous lesions, these develop as swelling and erythema that precede the skin induration and involve primarily fingers, hands, and face (24). Digital vasculopathy mainly manifests with Raynaud’s phenomenon, due to the associated changes in small blood vessel structure. In the advanced stage of vasculopathy, ischemic pain, and digital ulcerations can occur. The large majority of patients with SSc have gastrointestinal symptoms. These encompass dysphagia, cough due to gastroesophageal reflux, bloating, and constipation. In a few cases, they may have the most serious events such as gastrointestinal bleeding and anemia (23). Musculoskeletal manifestations include fatigue and stiffness of the fingers in the first stages of the disease, but arthralgia, tendonitis, joint contractures, and arthritis are also frequent (25). Pulmonary involvement can include pulmonary fibrosis and pulmonary vascular disease and manifests with non-productive cough and dyspnea (17).

Due to the heterogeneity of the disease in terms of clinical presentation, an international work involving the American College of Rheumatology (ACR) and the European Alliance of Associations for Rheumatism (EULAR) developed classification criteria considering clinical and laboratory parameters, with the aim of generating uniformity in disease detection and management (**Table 1**) (26). Importantly, the risk of death in patients with SSc is up to fourfold higher than in subjects taken from the general population and balanced for age and sex (27). The principal causes of death in patients with SSc are due to pulmonary fibrosis, pulmonary hypertension causing heart failure, kidney disease, and malignancy (27). The importance of such findings is even reinforced by the scarce results provided by previous randomized clinical trials around how to treat the disease, slow progression, and reduce future risk. This is particularly true with respect to the limited efficacy of immunosuppressive treatment in improving SSc prognosis and also testifies that the comprehension of the abovementioned immunologic mechanisms of disease does not fully explain the underlying cause of organ failure (28). Thus, reaching a reliable risk stratification and personalized treatment of the disease represents two key-points of clinical research around this topic. A big effort is still needed in this direction.

**Table 1:** The 2013 ACR–EULAR criteria for the classification of systemic sclerosis.

Item	Sub-item(s)	Weight/score
Skin thickening of the fingers of both hands extending proximal to the metacarpophalangeal joint *(Sufficient criterion)*	–	9
Skin thickening of the fingers *(Only count the higher score)*	Puffy fingers sclerodactyly of the fingers (distal to the metacarpophalangeal joints but proximal to the proximal interphalangeal joints)	2 4
Fingertip lesions *(Only count the higher score)*	Digital tip ulcers Fingertip pitting scars	2 3
Telangiectasia	–	2
Abnormal nailfold capillaries	–	2
Pulmonary arterial hypertension and/or interstitial lung disease *(Maximum score is 2)*	Pulmonary arterial hypertension Interstitial lung disease	2 2
Raynaud’s phenomenon	–	3
SSc-related autoantibodies (anticentromere, anti-topoisomerase I [anti-Scl-70], and anti-RNA polymerase III) *(Maximum score is 3)*	Anticentromere Anti-topoisomerase I Anti-RNA polymerase III	3

The total score is determined by adding the maximum score in each category. Patients with a total score of 9 or more are classified as having definite SSc. ACR, American College of Rheumatology; EULAR, European Alliance of Associations for Rheumatism; SSc, systemic sclerosis.

Kidney involvement in SSc is common. Clinical findings of altered kidney function (reduced epidermal growth factor receptor (eGFR) levels) or presence of kidney damage (increased urine protein excretion) can be found in half of the patients, albeit in part of these cases related to other concomitant causes or comorbidities (29, 30). The most severe disease manifestation, namely, the SRC, is not negligible and occurs in about 20% of cases (30). Regardless of the cause of kidney damage, a proportion of patients with SSc will progress to end-stage renal disease (ESRD), the most advanced stage of chronic kidney disease, which often requires the need of renal replacement therapies (RRTs) (31). Kidney transplantation (KT) is considered the ideal therapeutic option for ESRD patients, as it warrants a better survival as compared with dialysis (32, 33).

The aim of the present narrative review is to provide an overview of the role of RRT and KT in SSc patients, trying to point out the potential influence of KT on the prognosis of patients and the disease’s evolution.

## Kidney involvement in systemic sclerosis

SSc involves and damages the kidney in a non-trivial proportion of patients. What is important to discriminate is whether the disease causes the presence of abnormalities in kidney function (increased urine albumin excretion and/or decreased eGFR levels) or whether these abnormalities are due to concomitant conditions. However, regardless of the specific active mechanism of the disease, the presence of kidney abnormalities in SSc patients has been associated with a threefold increased risk of death (1). Thus, such findings have led physicians to include the assessment of kidney measures in the routine clinical follow-up of SSc patients (34). The most frequent kidney damage in SSc is the SRC, which also represents the most severe complication, the concomitant antineutrophil cytoplasmic antibody (ANCA)-associated vasculitis, and the forms with isolated kidney abnormalities (34). SRC develops in about 20% of cases of SSc with diffuse cutaneous disease, whereas it is rare in limited cutaneous disease (35, 36). The crucial point of the pathogenesis of SRC seems to be the injury of the arcuate and interlobular renal arteries with their thickening and proliferation (37). This leads to narrowing of the vascular lumen with ‘onion-skin’ hypertrophy. The histology does not provide pathognomonic signs of SRC, as these features can be detected in other conditions such as thrombotic thrombocytopenic purpura, hemolytic uremic syndrome, and chronic kidney transplant rejection; thus notwithstanding, a kidney biopsy can provide useful prognostic information in these patients (38). From the clinical perspective, SRC usually occurs in the first years after the onset of the disease and is characterized by acute kidney injury (rapid deterioration of renal function, which occurs in less than 1 month), severe arterial hypertension with signs of malignant hypertension such as retinal hemorrhages and exudates, and hypertensive encephalopathy and mild proteinuria at 24-h urine (usually 0.5 to 1.0 g) (36). Prognosis of this condition is often negative, with a very rapid (even in weeks) progression to ESRD. Hence, close monitoring and early detection are mandatory. Other than the diffuse skin SSc, other risk factors that predict the onset of SRC are the presence of RNA-polymerase III antibodies, the use of glucocorticoids, cyclosporine, and cardiovascular disease (heart failure or new onset of cardiac events) (39). Similarly, proper and prompt treatment is definitely important in patients with SRC. The cornerstone of the medical therapy of SRC consists of lowering and normalizing blood pressure levels. Albeit randomized clinical trials have not been conducted, there is sufficient evidence suggesting the efficacy of angiotensin-converting enzyme inhibitors (ACEis) in slowing the progression to ESRD and improving blood pressure control and patient survival (40, 41). Patients treated with ACEis and corticosteroids have a greater chance of not developing ESRD or discontinuing dialysis, and, in this case, they achieve survival rates similar to those of patients who do not experience SRC. Nevertheless, about 25%–50% of patients with SRC still have a bad prognosis, characterized by permanent dialysis and early death (1, 41–43). In general, the authors emphasize the need of an early diagnosis and early aggressive treatment with ACEis, especially in those patients presenting with relatively modest rises in blood pressures and/or creatinine. A close monitoring of blood pressure in patients with early diffuse cutaneous SSc and/or anti RNA polymerase antibodies is a good example of preventive measure. However, even if a small proportion of patients with limited disease will develop SRC, it is also relevant for them to monitor blood pressure and reach a tertiary care center in case it increases above 140–150/90 mmHg (41, 43).

ANCA-associated vasculitis has been detected in up to 9% of patients with SSc (44). It has been hypothesized that the SSc lesions predispose to the interaction between ANCAs and endothelial cells in the kidney, thus triggering the activation of kidney damage (1). In contrast to SRC, ANCA-associated vasculitis is more frequent in SSc patients with limited skin diffusion of disease, and the presentation of symptoms is less abrupt with mild hypertension, progressive decline of kidney function, and mild proteinuria. The presence of albuminuria is detectable in 15%–25% of patients and is mostly related to the vascular chronic lesions of SSc (45). Similarly, cases with isolated eGFR reduction are common. In these patients, the vascular lesions are predominant with glomerular hypofiltration (45). However, it has been shown that the presence of low eGFR is associated with a higher likelihood of developing pulmonary hypertension and heart failure, so it deserves meticulous attention (46).

## Renal replacement therapy in systemic sclerosis

In the last years, many studies have reported poor outcomes for patients with SSc and ESRD. The French REIN registry has shown that among 98 SSc patients dialyzed between 2001 and 2013, 81% had ESRD secondary to SRC. Their outcome was poor with a survival respectively of 75%, 55%, and 32% after 1, 3, and 5 years (47).

Another cohort study performed from the Dialysis and Transplant Australian and New Zealand registry showed, in patients with ESRD secondary to scleroderma, similar survival rates to those reported in the French study(72%, 55%, and 29% at 1, 2, and 5 years, respectively), which were significantly shorter than in patients with other causes of ESRD (48).

More recently, a European study performed from the ERA-EDTA registry analyzed the mortality from day 91 after the commencement of RRT in patients with scleroderma and compared these data with two matched control groups: a group of patients who started RRT because of diabetes and a group of patients who started this treatment due to other primary kidney diseases. Again, mortality was higher in the scleroderma group than in the other two groups, with a patient survival probability at 5 years of 38.9% for patients with scleroderma versus 46.0% for diabetic patients and 63.6% for patients with other kidney diseases (49).

Despite the limitations related to the registry-based nature of the data, with a lack of detailed information on patient characteristics, the aforementioned studies revealed that, among the possible causes of death, cardiovascular events and particularly myocardial infarction, were less common in ESRD scleroderma patients than in ESRD patients due to other causes. However, the number of unknown or miscellaneous causes of death was higher in the scleroderma group, revealing not only the difficulty of drawing any conclusion but also the complexity of this disease and patients’ management (48, 49).

The vascular access challenge in patients with scleroderma is significant because of the extensive vascular damage that characterized this pathology (50). The difficulty of fabricating a functioning arteriovenous fistula and the consequences of altering the vascularization in the relevant districts are described in a case report of Quan, which is presented the new onset of severe Raynaud’s phenomenon following capillary abnormalities, skin thickening, and finger deformity, after the formation of a fistula (51). Similarly, peritoneal dialysis (PD) is not devoid of any issues. The most severe complication of peritoneal dialysis is encapsulating peritoneal sclerosis (EPS), characterized by a progressive inflammatory process causing viscera constriction, promoted by fibrotic and angiogenic factors that are in common with systemic sclerosis pathogenesis (52). From this point of view, the idea of stimulating or accelerating a potentially life-threatening process is worrying.

Thus, the optimal dialytic option for patients with ESRD due to SSc is still uncertain. In the European cohort, patients with scleroderma were less likely to be treated with PD than hemodialysis (HD) when compared with the matched control groups, while data from the Australian and New Zealand registry showed that the use of PD was more common in patients with scleroderma than in patients with other causes of ESRD, maybe due to poor vascular access or to a propensity of clinicians to put scleroderma patients on PD because of the possible recovery of renal function (48, 49).

Studies comparing the outcome of SRC patients performing HD or PD are lacking, and the choice of dialytic option for these patients is still dependent on the clinician’s experience.

## The recovery of renal function

After the introduction of ACEis, the recovery rate has been analyzed in many retrospective or prospective reports. In a prospective study published in 2000, 23.4% of SRC patients requiring dialysis were able to discontinue a median time of 11 months (range 1–34 months) (53). In addition, a more recent retrospective case series including 110 patients with SRC treated with ACEis reported that a similar rate of patients could discontinue dialysis with a mean of 8 months and for up to 3 years. In this study, the poor renal outcome was associated with lower blood pressure at presentation and higher age, but not with corticosteroid use, microangiopathic hemolytic anemia, or antibody profile (43). Conversely, the study from the Australian and New Zealand registry reported that only 10% of patients recovered sufficient renal function to discontinue dialysis, with the recovery occurring in the first 12–18 months (mean 14.1 months) after dialysis initiation. However, the data analysis reveals that this low recovery rate might be due to the exclusion of patients with very early renal functional recovery (less than 3 months after dialysis initiation) (48). The same renal recovery rate (7.6%) has been reported also by the European cohort study performed by Hruskova et al. In this paper, the dialysis discontinuation rate has been compared between SSc patients and patients with other etiologies of ESRD, revealing a higher recovery rate in the first group. These data were expected considering that this event is even more frequent among ESRD due to other autoimmune diseases than other primary renal diseases (49).

## The timing of kidney transplantation

The optimal care of patients with ESRD due to scleroderma remains challenging. The survival of these patients following the initiation of dialysis is worse when compared to the overall dialysis population. For this reason, KT has been progressively taken into consideration with the expectation of conferring a survival advantage.

The timing of KT in these patients is still a matter of debate. The first question is how long to delay transplantation after dialysis initiation, considering the possibility of renal function improvement. Depending on the results of different studies, approximately one-third of patients are able to discontinue dialysis with a median time of 11–14 months. Considering this appreciable incidence of recovery of dialysis-independent renal function, the clinician should be circumspect about proceeding with KT during this early period (48). However, this decision should vary on a case-by-case basis. A renal biopsy might be useful to determine whether the recovery of renal function is possible (39). However, patients without any evidence of an improvement in renal function after 12 months from dialysis initiation might be considered for transplantation in order to gain faster improvement of expectation and quality of life (54). The second question that should be taken into consideration before KT is the evaluation of the disease activity. Clinicians should perform a critical evaluation of the patients’ clinical status, in terms of blood pressure control, extrarenal disease manifestations, and comorbidities (8, 9). Some case reports showed that the administration of endothelin-1 antagonists (i.e., bosentan) may ameliorate the outcome of patients with SRC who were experiencing worsening renal function and rapid necessity of RRT despite the use of ACEis (55, 56). Recently, a phase II randomized placebo-controlled trial reported the possible role of a highly selected endothelin-A antagonist, zibotentan, in stabilizing the eGFR trend, without a rebound of hypertension after the interruption of the drug (57). Thus, even if the number of cases treated with endothelin-1 antagonists is still too small to draw any conclusion, this therapeutic option could be taken into consideration in facilitating renal recovery after SRC and before evaluating patients for KT.

Finally, many interesting targets are under evaluation for the treatment of SSc: the CD28–CD80/86 pathway blocked by abatacept, which is able to suppress T-cell activation; the CCL24 inhibitors that reduce profibrotic activity; and monoclonal antibodies that inhibit tumor necrosis factor (TNF), TGF-beta, the B-cell activating factor (BAFF), and IL-6. Further investigations remain necessary in order to identify the more effective drug able to treat kidney manifestations of the disease (58).

## Graft, disease, and patient outcomes after kidney transplantation

The first KT was performed on a patient with SSc and was described by Richardson in 1973. In this case, the patient had an SRC with stable extrarenal manifestations. He was treated with bilateral nephrectomy to achieve optimal control of blood pressure. After KT, blood pressure and renal function were normal, and also arthralgia, Raynaud phenomenon, and skin lesions were ameliorated (59).

During the following years, other similar cases were reported in the literature, with different results regarding graft outcome and risk of disease recurrence. In 2005, Pham et al. reviewed all reported cases of renal transplantation in scleroderma patients and tried to identify predictors for graft SRC. They found that among patients who experienced disease recurrence, all of them had developed ESRD earlier after the onset of SRC in the native kidneys (within a year), while patients without SRC recurrence had slower damage in their native kidneys. In addition, although data were incomplete in some cases, in two patients, graft SRC was preceded by anemia; in one patient, graft SRC was preceded by pericardial effusion; and in two cases, there was a worsening in skin tightening (60–67).

In the same paper, the authors also described retrospective data about 260 patients with SSc who underwent KT, obtained from the United Network for Organ Sharing (UNOS) database. They revealed that 28.8% of them experienced graft loss with five (6.7%) due to disease recurrence. The time from KT to graft loss was highly variable at 70, 117, 131, 645, and 802 days. Other cases of graft loss were infection (2.7%), thrombosis (4%), primary failure (8%), acute rejection (16%), chronic rejection (30.7%), and other unreported causes (32%). The authors conclude that the risk of recurrent disease after transplantation was previously reported as higher (from 20% to 50%) probably because of an overreport of more difficult cases, while UNOS data, which revealed a smaller recurrence rate of 1.9%, may underreport this condition, considering the 32% lacking causes of graft loss (60).

More recently, in 2017, Bertrand et al. reported their multicenter French study, including 36 SSc patients who underwent KT between 1987 and 2013. They observed seven graft loss: one of them due to SRC, four secondary to antibody-mediated rejection, one due to a renal cell carcinoma of the graft, and one because of urinary septic shock. Two patients experienced kidney disease recurrence without graft loss. Death-censored graft survival was 97.2% after 1 and 3 years and 92.8% after 5 years, comparable to that of the global French cohort of kidney transplantation performed in a similar period. Patient survival was also very good: 100%, 90.3%, and 82.5% at 1, 3, and 5 years, respectively. The authors conclude that their data about graft and patient outcomes are better than previously reported, probably due to the improvement of KT management, the immunosuppressive therapy for rejection, and SSc during these last years (47).

A similar good outcome in terms of graft and patients’ survival was reported also in the study performed by Hruskova et al. in 2019, which included 57 patients who underwent KT between 2002 and 2013. They found a 5-year graft survival rate of 72.4%, but, particularly, they found that patients and graft survival after KT did not differ between patients with SSc and those with other primary kidney diseases, suggesting that KT may be the optimal therapeutic option for these patients, especially those without major comorbid conditions (49).

The literature review about the outcome of KT in SSc is summarized in **Table 2**.

**Table 2:** Overview of studies reporting the outcome of kidney transplantation in patients with end-stage renal disease due to systemic sclerosis.

Author	Study and year of publication	No. KTR	Mean age at KT	Patients survival			Non-death-censored graft survival			Recurrence of SRC	No. graft loss	Death with a functioning graft	Mean follow-up
				1 year	2 years	3 years	1 year	2 years	3 years				
Richardson ^36^	Case report, 1973	1	41	100%	100%	100%	100%	100%	100%	n/a	n/a	n/a	88 months
Merino et al. ^40^	Case report and review of literature, 1977	1	41	100%	n/a	n/a	0%	0%	0%	100%	1 (100%)	0%	n/a
Caplin et al.^43^	Case report, 1999	1	61	100%	100%	n/a	100%	0%	0%	100%	1 (100%)	0%	n/a
Gibney et al. ^35^	Retrospective, 2004	142	52 ± 10	90.1%	n/a	79.5%	68%	n/a	60.3%	4.8%	63 (44.4%)	30 (21.1%)	83 months
Chang et al. ^44^	Case reports, UNOS database, 1999	86	50.4	n/a	n/a	n/a	62%	60%	57%	3.48%	38 (44%)		60 months
Pham et al. ^37^	Case report and review literature, UNOS database, 2005	260	n/a	89.79%	83.94%	81.09%	78.7%	73.3%	68.59%	1.9%	75 (28.8%)	n/a	120 months
Siva et al.^32^	ANZDATA registry, 2011	16 deceased donors	47.7 ± 10.3	n/a	n/a	n/a	78%	n/a	28%	n/a	n/a	n/a	120 months
		6 living donors		n/a	n/a	n/a	100%	100%	100%
Bertrand et al.^31^	Retrospective multicenter, 2017	36	52.9 (27.7–75.5)	100%	n/a	90.3%	97.2%	n/a	87.8%	8.3%	7 (19.4%)	7 (19.4%)	180 months
Hruskova et al.^33^	Retrospective case-control multicenter, ERA-EDTA registry, 2018	57	n/a	90.7%	90.7%	90.7%	89.6%	85.8%	85.8%	n/a	n/a	n/a	60 months

KTR, kidney transplant recipients; KT, kidney transplantation; SRC, scleroderma renal crisis.

## Immunosuppressive therapy after kidney transplantation

There is no consensus on the role of immunosuppressive therapy after KT in preventing allograft rejection and improving systemic manifestations in patients with SSc. In fact, due to the small number of studies reporting the management of SSc patients who underwent KT, an ideal immunosuppressive protocol has not been established yet. As in patients with other causes of ESRD, the most common induction therapy includes high doses of steroids and anti-thymocyte globulin or anti-interleukin-2 receptors, while there is more debate about maintenance therapy (47). Previous studies postulated that calcineurin inhibitors (CNIs) should be avoided in the immunosuppressive regimen of these patients because their endothelial toxicity could worsen the vascular damage implicated in the pathogenesis of SSc (68). However, in their study, Bertrand et al. included many KT patients treated with CNIs and did not find any noticeable adverse events (47). Glucocorticoids have classically been considered a risk factor for SRC. Thus, the maintenance regimens provide that they should be rapidly withdrawn (69). *In vitro* studies, experimental animal models, and early clinical trials have demonstrated the potential efficacy of mTOR inhibition in SSc management through decreasing collagen production from dermal fibroblasts (70, 71). However, hypertension, edema, and increased levels of creatinine and proteinuria are more common with these drugs, and consistent evidence-based recommendations are still lacking. However, immunosuppressive treatment with mycophenolate mofetil is a cornerstone for the treatment of lung and cutaneous involvement in patients with SSc. Thus, in KT after SSc, patients should be treated with the conventional immunosuppressive therapy including CNIs and mycophenolate mofetil, while mTOR inhibitors should be taken into consideration in selected cases (72).

Even if there are no data regarding the relapse of SRC in KT patients who had stopped ACEi therapy, all of these patients should receive ACEis due to their renoprotective and antihypertensive effects. A case report has described the recurrence of SRC after KT in a patient who was switched from ACEis to losartan (73). Again, no consistent data are available to contraindicate the use of angiotensin receptor blockers in these patients.

## The evolution of extrarenal involvement after kidney transplantation

Few data are available about the evolution of extrarenal disease manifestations in SSc patients after KT. Gibney et al. reported for the first time the modification of skin lesions in four patients who underwent renal transplantation, analyzed through the Rodnan score. Each patient had a decline in skin scores with an average reduction of 61%. However, two of them experienced an early renal recurrent disease (54).

More extensive data about extrarenal manifestations at the time and after transplantation have been provided by Bertrand et al. In particular, with the limitations due to the retrospective nature of the study, they found a high rate of worsening in cardiac and gastrointestinal manifestations, while an improvement in extrarenal manifestations was rarer. Furthermore, pulmonary involvement of SSc (including interstitial lung disease and pulmonary hypertension), even if rare in those patients who develop SRC, was found to be a post-transplant independent risk factor for death. The authors conclude that close monitoring of extrarenal involvement is necessary before and after KT and that special caution should be paid to finding any lung disease before the transplantation, which could constitute a contraindication for KT (47).

**Figure 1:**
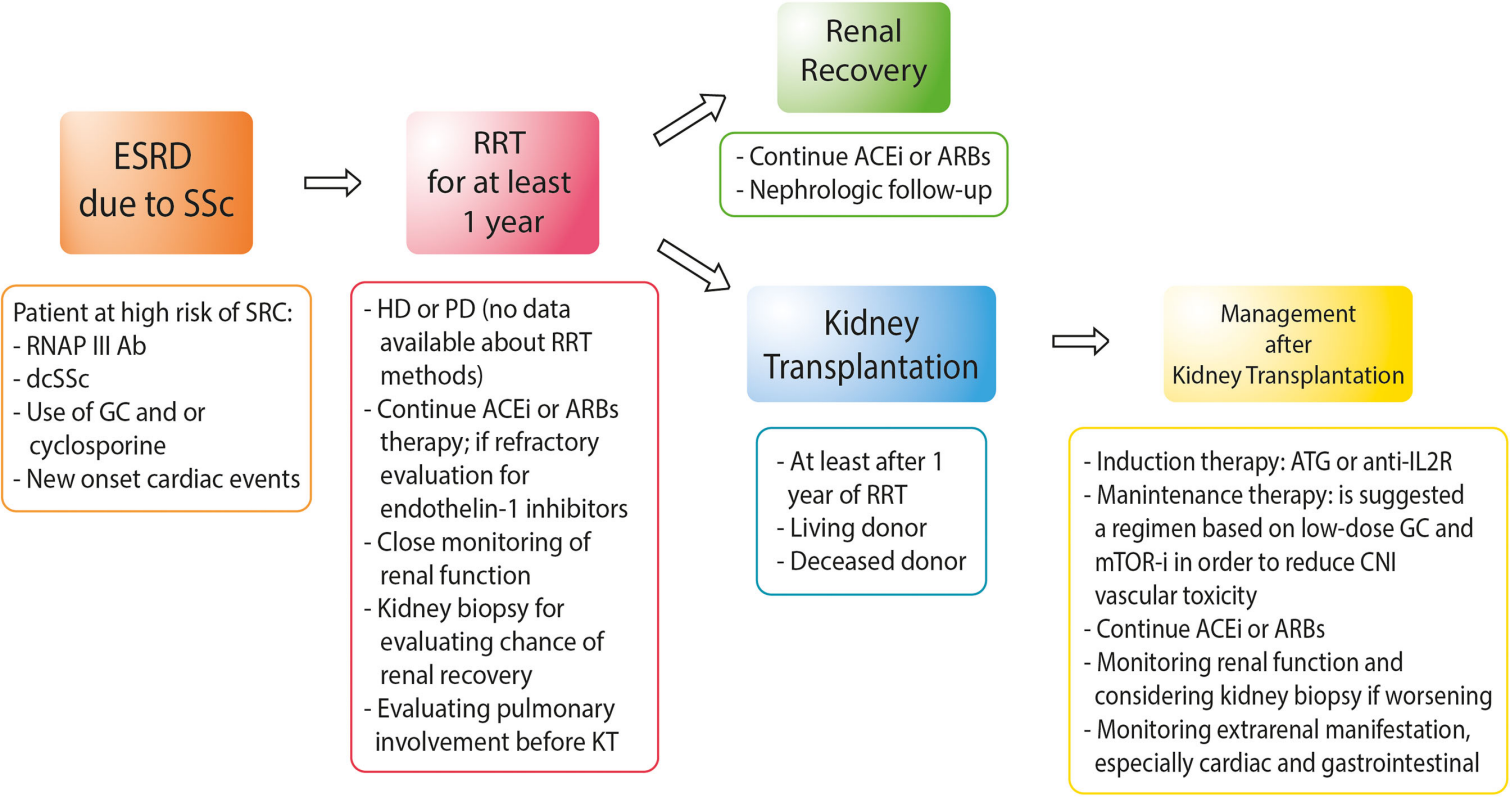
Approach to the management of patients with end-stage renal disease due to systemic sclerosis. RNAP III Ab, anti-RNA polymerase III antibody; dcSSc, diffuse cutaneous systemic sclerosis; GC, glucocorticoids; HD, hemodialysis; PD, peritoneal dialysis; ACEis, angiotensin-converting enzyme inhibitors; ARBs, angiotensin receptor blockers; ATG, anti-thymocyte globulin; anti-IL2R, anti-interleukin-2 receptors; mTOR-i, mTOR inhibitors.

**Figure 1** shows the suggested management of SSc patients from ESRD to KT.

## Conclusions

SSc is a multisystem disease with a relatively frequent different grade of kidney involvement. The condition of ESRD is a risk factor for a bad prognosis. The most common cause of ESRD in these patients is SRC, a rare but life-threatening complication characterized by the new onset, often symptomatic hypertension, rising serum creatinine levels, and/or oligoanuria. The advent of the ACEis in the 1980s was associated with a significant improvement in renal function and patients’ survival. However, about 20% of SRC patients maintain dialysis dependence, and this condition is associated with high mortality. Even if randomized studies are lacking and data about KT in SSc patients are actually limited to case series or retrospective cohort studies, this option appears to confer a gain in survival, with graft and patient outcomes as good as those reported in KT patients due to other kidney diseases. Also, the timing of KT has not been established yet; however, a reasonable option could be waiting for 1 year after dialysis begins and, eventually, performing a kidney biopsy, before the transplantation.

Efforts should be made in order to recognize parameters able to predict the disease recurrence after KT, which is a rare but frequent cause of graft loss. However, other differential diagnoses causing deterioration of kidney function, including rejection or thrombotic microangiopathy, should always be ruled out. Very few data are available about the modifications of extrarenal manifestations in SSc patients with KT. Further studies are warranted to determine if the resolution of the uremic status could induce amelioration of the involvement of other organs and, in this case, to identify the immunological or metabolic basis of this process.

## Author Contributions

FM, GC contributed to conception and design of the study. FM, MP, GC and GM reviewed the literature. FM, MP, GC and GLM wrote the first draft of the manuscript. FM, MP, GC, SL, VCO, CB, MB, VG, VCU and GM wrote sections of the manuscript. All authors contributed to manuscript revision, read, and approved the submitted version

## Funding

This study was supported by research fundings from the Italian Health Ministry.

## Conflict of Interest

The authors declare that the research was conducted in the absence of any commercial or financial relationships that could be construed as a potential conflict of interest.

## Publisher’s Note

All claims expressed in this article are solely those of the authors and do not necessarily represent those of their affiliated organizations, or those of the publisher, the editors and the reviewers. Any product that may be evaluated in this article, or claim that may be made by its manufacturer, is not guaranteed or endorsed by the publisher.
